# Does declining income caused by the COVID-19 pandemic affect Chinese individuals’ future risky decision-making and intertemporal choices? A construal level perspective

**DOI:** 10.3389/fpsyg.2025.1584337

**Published:** 2025-06-20

**Authors:** Yifei Hua, Jiaxin Mi

**Affiliations:** ^1^School of Public Policy and Management, China University of Mining and Technology, Xuzhou, China; ^2^School of Safety Engineering, China University of Mining and Technology, Xuzhou, China

**Keywords:** savings and consumption behavior, individual difference, conditional process model, COVID-19 pandemic, behavioral responses

## Abstract

In order to evaluate how residents choose savings and consumption behavior within the context of the income changes, which resulted from the COVID-19 pandemic, this paper constructed a conditional process model to study the impact of residents’ declining income on their low risk savings behavior and advanced consumption behavior from a cognitive perspective. We found that residents with declining income preferred low risk savings and advanced consumption behavior; residents with lower incomes, whose original income was higher, were more likely to be influenced by anxiety about perceived emergency supply shortages. The moderating role of social trust emerged only after supply shortage anxiety mediated the effect, and this moderation differed significantly based on construal level. This paper explored the impact of declining income and the emotional fluctuations that resulted from the COVID-19 epidemic on the differential choice of residents’ savings and consumption behavior. It can provide microeconomic guidance for similar emergencies in the future.

## 1 Introduction

The outbreak of COVID-19 in early 2020 presented challenges at a national level (Tisdell, 2020; Geirdal et al., 2021), and the economic downturn and financial burdens which resulted from the epidemic will also impact upon the daily life of residents. The long-lasting and extensive impact will undoubtedly intensify the negative emotions of residents (Zhang et al., 2019; Daniali et al., 2023; Motta and Benegal, 2023). In addition to the direct impact of this epidemic, limited industrial development will also indirectly result in a decline in employees’ incomes in some industries. Perceptions regarding the impact of declining incomes will further increase feelings of emotional vulnerability among those affected, particularly in terms of decision-making regarding household saving and consumption behavior (HSCB). As the foundation of national economic activities, HSCB is concerned with transforming a country’s economic mode of development. Therefore, it is necessary to explore the impact of Chinese families’ declining income on HSCB from the perspective of residents’ emotional attitudes during the epidemic.

According to Cognitive Behavior Theory (Steiger et al., 2019; Wang and Guo, 2024), human behavior can be guided by the dynamic neuropsychological process of emotion. Is this superimposed negative sentiment limited to the period of the epidemic, or will it also have an impact on residents’ HSCB when the epidemic has subsided? From the perspective of Construal-level theory, different levels of understanding among residents will affect or even change their behavioral decision-making process (Brügger et al., 2016; Reczek et al., 2018; Zhang et al., 2024). If income levels, both before and after the epidemic, are used as the main scale by which to classify residents, will the samples with different levels of income decline reveal different HSCB patterns?

Scholars from the fields of social emergency management and those concerned with investigating the mood fluctuations of residents are widely concerned with the issue of public emergencies (Abeysinghe et al., 2017). However, few research studies have examined family economic behavior after a public emergency, and there is a notable lack of research on differences in HSCB patterns as a result of income or industry differences, or residents’ emotional fluctuations. In view of the important influence of family economic behavior on the stable operation of the national economy, this paper develops a conditional process model based on construal-level theory.

This study aims to advance research regarding the impact of declining income on residents’ HSCB after the epidemic, to examine differences in behavior which result from a decline in income, and identify how declining income impacts upon construal level. Thus, this study can provide theoretical support and practical guidance from a micro-level perspective, which can also provide a reference for emergency management in the event that similar social emergencies occur in the future.

### 1.1 Construal-level theory and behavior choice

#### 1.1.1 Construal-level theory

Construal-level theory (CLT) was first developed by Liberman from the Time Interpretation Theory, which is a cognitive orientation theory. According to the theory of time interpretation, temporal distance can affect people’s value judgments. In line with the construal-level, it can be divided into the following: Under the high Construal level (CL), the value weight increases with the passage of time; under the low CL, the greater the temporal distance, the lower the value weight (Trope and Liberman, 2010; Xu et al., 2024b). That is to say, compared with the long-term future, the feasibility of short-term target events has a greater impact on behavioral decision-making than the acceptability (Liberman and Trope, 1998).

By applying CLT, it can be understood that people’s behavior and decision-making are dependent upon their psychological representation of the target event (i.e., a high/low-level construal). The individual CL can be divided into the personality CL which is based on individual thinking habits, and the situational CL which is based on the occurrence of specific situations. In other words, the difference in the spatial, temporal and social distance, and the probability that the event will occur, will generate different CLs among residents (Kim and John, 2008; Li et al., 2024). In comparison, a decision-making situation is key to eliciting different cognitive tendencies. In general, these four basic psychological distances share the same anchor point (Martina et al., 2015; Chen and He, 2011). Fiedler et al. (2012) used these four basic psychological distances to evaluate the same goal and found that they regularly show a significant and positive correlation. In addition, in the process of applying CLT, some scholars introduced new concepts, such as “information distance,” “emotional distance” and “experience distance “to classify the CLs (Dhar and Kim, 2007). Therefore, CLT provides a comprehensive theoretical framework for understanding the change in behavioral preferences from different perspectives.

#### 1.1.2 Risky choices and intertemporal choice behavior

Intertemporal choice refers to decisions individuals make through deliberate evaluation of values across distinct time horizons (Frederick et al., 2002). Many scholars have pointed that there are similarities between time and probability, and risky decision-making and intertemporal choice (Green and Myerson, 1996). From the perspective of risk, the smaller the probability of obtaining the target result, the longer the waiting time, so as to stimulate the different psychological distance (Chen and He, 2011). When considered in terms of CLT, it can be understood as follows: The greater the psychological distance, the more likely it is that an individual will delay their intertemporal choice, and the more likely they are to choose a high risk option during the risk selection process.

In addition to psychological distance, an individual’s emotional state also affects their preferred choice (Schwartz et al., 2018). In the case of an event such as a sudden disaster, Bchir and Willinger (2013) and Cassar et al. (2017) both found that the impact of the affected groups’ perceptions diminished the amount of patience they had to wait to benefit from something in the future, so they showed a preference for the present vested interests (benefits will be available in the near future) (Siddiqui et al., 2018).

### 1.2 The function of social trust in emergencies

Frederick et al. (2002) summarized more than 40 literature publications on risk behavior and cross-period behavior, and found that different conclusions were reached about the time discount rate due to different decision-making situations. In view of this, we added social trust as a moderating variable to specify the decision-making situation. In view of this, we added social trust as a moderating variable to specify the decision-making situation. As an epidemic disaster, the COVID-19 pandemic is a typical public health emergency. This means that the epidemic situation is not only a public health problem, but a social problem that has a profound impact on the lives of residents, which has therefore presented an unexpected challenge for social governance (Zhang et al., 2019; Yuan et al., 2022). Therefore, we choose Medical trust (Evans et al., 2019; Leung et al., 2022), Media trust (Shan et al., 2019; Shahbazi and Bunker, 2024) and Community trust (Schnall et al., 2018; Gilmore et al., 2020) as the compositions of the social trust.

### 1.3 Anxiety about supply shortages and negative emotions during the epidemic

Early deployment and reasonable planning in regard to supplies can enhance the efficiency with which public emergencies are managed (Sabbaghtorkan et al., 2020; Zhang and Wang, 2023). Although most countries are aware of this problem, there will always be a shortage of supplies for a short period of time, the most prominent and influential of which include medical supplies and essential emergency supplies (Li et al., 2021; Xu et al., 2024a). The former is related to the treatment of patients and public protection (Ochi et al., 2020), while the latter is related to the basic daily living and survival needs of all people. Abeysinghe et al. (2017) even pointed out that a shortage of basic supplies will also affect the ability of medical staff to care for patients. In addition to the above effects, a shortage of supplies will further intensify the public’s emotional vulnerability (Al-Dahash et al., 2019; Zhao et al., 2020). As a dynamic neuropsychological process, emotions also lead people to make corresponding behaviors (Steiger et al., 2019).

Many scholars have highlighted that negative emotions can increase people’s sensitivity to time and distance, which makes people more inclined to meet current needs rather than longer-term goals, and they are more conservative in their risk selection (Lerner and Keltner, 2001; Janssen et al., 2021; Fan et al., 2023). That is to say, panic, anxiety and other negative emotions, which arise in response to a lack of supplies, will diminish people’s capacity to exert self-control with regard to their pursuit of long-term goals that have the potential to maximize their benefits, and they therefore make short-sighted decisions (Mullainathan et al., 2013).

### 1.4 Literature summary and hypothesis proposal

Based on the analysis of the above literature, we found that the contribution of existing literature can be summarized as follows: (1) Studies establish that both psychological distance and emotional states shape risky and intertemporal choices. While psychological distance promotes delayed gratification and risk-seeking, sudden disasters like pandemics amplify negative emotions, driving short-term preferences. (2) Research highlights social trust’s role in crisis decision-making, particularly medical, media, and community trust. These dimensions influence risk perception and compliance, though findings vary across cultural contexts. (3) Resource shortages exacerbate emotional vulnerability, which heightens present bias and risk aversion. This link is critical for understanding crisis-driven impulsivity.

However, current research still has some critical research gaps: (1) Theoretical integration of competing mechanisms: existing models isolate psychological distance (rational) and emotion-driven pathways, neglecting their interaction during prolonged crises. (2) Multidimensional trust dynamics: while trust dimensions (medical/media/community) are recognized, their synergistic or antagonistic effects remain unexplored. For example, high medical trust may buffer anxiety, but excessive media trust could trigger information overload, yet no study quantifies these trade-offs.

In summary, the occurrence of a situation involving behavior, individual emotion and behavioral choice form a reasonable causal chain of behavioral choice. The existing literature focuses on the traditional concept of psychological distance and less on exploring experience distance when applying CLT. Furthermore, most of the research studies on sudden public health events (SPHE) are relatively one-sided, and few studies exist on the multi-dimensional social impact on residents’ behavioral choices, especially HSCB, which means that there is no reference for the impact of SPHEs on HSCB in the future.

**Figure 1:**
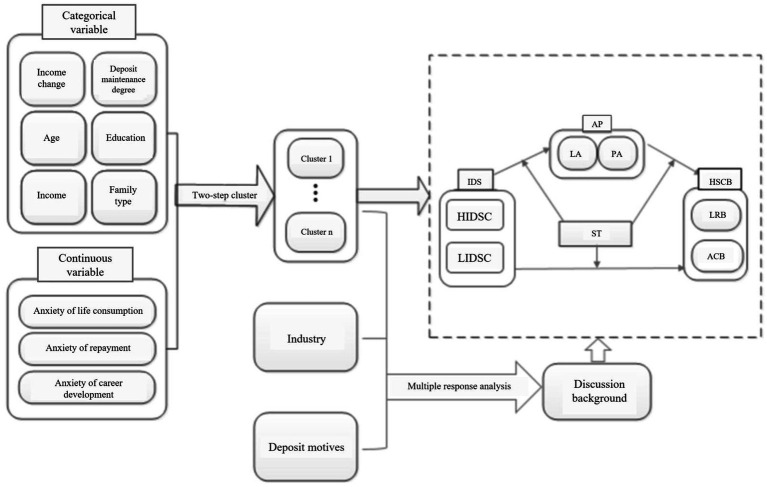
Technology roadmap.

By bridging theoretical fragmentation and contextual specificity, this study used variables related to family income as the breakthrough point, and took the COVID-19 pandemic as the situational context. The research design of this paper is shown in Figure 1, and the abbreviations for the main variables are illustrated in Appendix 1.

(1) First, in the two-step cluster analysis, categorical variables comprised income-related factors and demographic characteristics, while continuous variables included three anxiety dimensions induced by income changes: consumption anxiety, repayment anxiety, and career development anxiety.
(2) The cluster results, the employment industry of family members, and the household deposit motives were analyzed by referring to multiple responses. The results of the analysis formed the background to the discussion section of this paper.
(3) Then, taking the cluster results as the basis by which to divide the samples, the samples were classified into HIDSC and LIDSC for the conditional process tests. Among them, the independent variable was classified as a sample serial number; the intermediary variables were LA and PA; the moderating variable included medical trust, media trust and community trust; the dependent variables were low risk saving behavior (LRB) and advanced consumption behavior (ACB).

According to the literature review and the specific situational context of this study, this paper puts forward the following assumptions:

*H1:* Compared with residents with HIDSC, residents with LIDSC are more inclined to demonstrate in LRB and ACB.

*H2:* For residents with LIDSC, AP has a stronger influence on the LRB and ACB.

*H3:* Compared with residents with HIDSC, social trust can more effectively alleviate the impact of AP on residents with LIDSC in LRB and ACB.

*H3-1:* Social trust can more effectively regulate the role path of residents with LIDSC on AP.

*H3-2:* Social trust can more effectively regulate the direct path of residents with LIDSC on LRB and ACB.

*H3-3:* Social trust can more effectively regulate LIDSC residents’ LRB and ACB choice.

## 2 Research design and methods

### 2.1 Research tools

#### 2.1.1 Income decline shock perception scale

The income decline anxiety scale was used to measure residents’ perceived anxiety caused by income changes during the epidemic period. The scale was divided into three dimensions as follows: Anxiety about current consumption, anxiety about repayment, and anxiety about career development. A five-level Likert scales was used to measure responses and scores ranged from 1 (completely inconsistent) to 5 (completely consistent). The higher the score, the higher the level of perceived anxiety associated with declining income.

#### 2.1.2 Social trust scale

The measurement of social trust was divided into two levels which consisted of four dimensions: The national level consisted of medical trust and media trust; the community level consisted of community trust and community evaluation. The items were measured using a five-level Likert scales scale and scores ranged from 1 (totally inconsistent) to 5 (totally consistent). The higher the score, the higher the level of social trust.

#### 2.1.3 Supplies anxiety perception scale

In view of the impact of the supply quantity and quality control of materials on the psychology of residents during the epidemic, we developed the supplies anxiety perception scale. It consists of two parts, i.e., perceived anxiety about basic daily living supplies and perceived anxiety about prevention and control supplies. A five-level Likert scales was used to measure responses which were scored from 1 (totally inconsistent) to 5 (completely consistent). The higher the score, the more serious the residents’ perceived anxiety about supply shortages.

#### 2.1.4 Behavioral choice scale

In the scale, behavioral choice was divided into two dimensions, i.e., low risk saving behavior (LRB) and advanced consumption behavior (ACB). The items in each dimension are related to saving behavior and financing behavior; and repayment behavior and consumption behavior, respectively. A five-level Likert scales was used to measure responses and scores ranged from 1 (totally inconsistent) to 5 (totally consistent). The higher the score, the more residents prefer this type of behavior.

### 2.2 Data collection

In this study, 1,436 questionnaires were distributed using the Internet. After screening the data quality of the questionnaire, 1,352 questionnaires were deemed valid and these constituted the research samples. The research protocol received Ethical approval from the Academic Committee, and all participants were provided with Informed consent before participation. The effective recovery rate was 94.15%. The scope of the distribution covered 32 provincial administrative regions of China except Hong Kong, Macao and Taiwan. The demographic variables of valid questionnaire samples are shown in Table 1.

**Table 1:** Descriptive analysis of population variables.

Demographic variable		Number	Percentage
Gender	Male	702	51.92%
	Female	650	48.07%
Age	Under 18	1	0.07%
	18–25	471	34.83%
	26–30	191	14.13%
	31–40	218	16.12%
	41–50	230	17.01%
	51–60	218	16.12%
	Above 60	23	1.70%
Education level	Upper Secondary or under	88	6.50%
	Sub-degree	152	11.24%
	Bachelor’s Degree	724	53.55%
	Master’s Degree or above	388	28.87%
Monthly income (RMB)	Under 3,000	245	18.12%
	3,000–5,000	241	17.83%
	5,000–8,000	275	20.34%
	8,000–11,000	255	18.86%
	11,000–20,000	226	16.72%
	20,000–50,000	94	2.51%
	Above 50,000	16	1.18%
Family type	Living alone	183	13.54%
	Married^1^	226	16.72%
	Married (Kids)^2^	587	43.42%
	3 or 4 generations	274	20.27%
	Other	82	6.07%

1 Married but do not have children or children do not live together.

2 Married and live with children.

It can be seen that the gender distribution of the respondents was balanced; approximately 98% of the respondents were aged 18–60 years old, and the education level mostly included those with an undergraduate level education or above, which basically represents the backbone of the society. In addition to the scale of monthly income of more than 20,000 (yuan), the sample distribution of other monthly income ranges was relatively average; the family type mostly included married couples and fewer families living alone. Among them, the distribution of gender and family types is basically consistent with the data released in the seventh national census bulletin of China (National Bureau of Statistics in China (NBSC), 2021).

### 2.3 Data statistics

In this paper, SPSS version 22.0 was used to preprocess the data. First, the reliability and validity of the questionnaire design were tested to ensure the validity of each item. The key variables were then analyzed by carrying out a descriptive statistical analysis, Pearson’s correlation analysis and common method deviation test. After completion of data pre-testing, the Two-step clustering of the main variables related to residents’ income was firstly carried out, and all samples were classified according to the clustering results. The Multiple response analysis of the classification results related to occupation and original saving behavior was then carried out to determine the background of the discussion. Finally, the Process Macro plug-ins was used to test the Conditional process model presented in this paper.

## 3 Results

### 3.1 Reliability and validity test

Before the formal empirical study, the reliability and validity of the sample data were tested to ensure the rationality and validity of the questionnaire design. The test results of reliability and validity are shown in Table 2.

**Table 2:** Reliability test, KMO value test and Bartlett Spherical test of the questionnaire.

Variables		Item	Coefficient	Mean value	Inter-item correlation	KMO value	Bartlett’s Test of Sphericity		
							Approx. Chi-Square	df	Sig.
Social trust	McT	2	0.810	4.261	0.681	0.736	3,572.143	6	0.000
	MeT	2	0.616	3.900	0.445				
	CT	2	0.922	4.084	0.855				
Anxiety perception	LA	4	0.773	3.254	0.446–0.854	0.722	2,146.865	6	0.000
	PA	4	0.750	3.051	0.323–0.799				
Behavior choice	LRB	4	0.822	3.362	0.463–0.646	0.889	7,791.217	45	0.000
	ACB	4	0.786	2.887	0.357–0.575				

According to the data in Table 2, the Cronbach’s coefficients of all of the variables were above 0.6. Besides MeT, the coefficients of all of the other variables were above 0.75, and the coefficients of CT, CE and ADB were more than 0.9, which demonstrated that the questionnaire design has good rationality. The correlation coefficient between items and population was above 0.3, which also showed that the item design related to each variable is logical and can pass the test.

The validity test results showed that the KMO values of the three groups of variables were all above 0.7. Bartlett’s spherical values were large and the significance was.000, which means that the statistical value was significant. In addition, the correlation coefficient matrix was significantly different from the unit matrix, which showed that the structural design of the questionnaire was effective. In conclusion, the test results of the reliability and validity of the questionnaire showed that the item design in the questionnaire was reasonable and effective, and the collected data could be used for empirical analysis.

### 3.2 Two-step cluster

#### 3.2.1 Clustering results

Due to the large number of individual difference variables in the questionnaire, and in order to ensure an adequate discussion of typical samples, we aimed to reduce all of the samples to several representative categories of individual income differences by using two-step cluster analysis. Among them, the categorical variables included age, education level, income level, family type, income changes after the epidemic, and deposit maintenance. The continuous variables included consumption anxiety, repayment anxiety and career development anxiety as a result of income changes.

Based on a comprehensive evaluation of each index, we selected a row with a relatively small BIC and a relatively large absolute value BIC Change and Ratio of Distance Measures. It can be found that the clustering effect was optimal when all of the samples were divided into three categories. After inputting nine variables, all of the samples were finally divided into three categories, and the classification effect is “Fair.” Therefore, the classification result is effective.

#### 3.2.2 Analysis of the characteristics of clustering results

**Figure 2:**
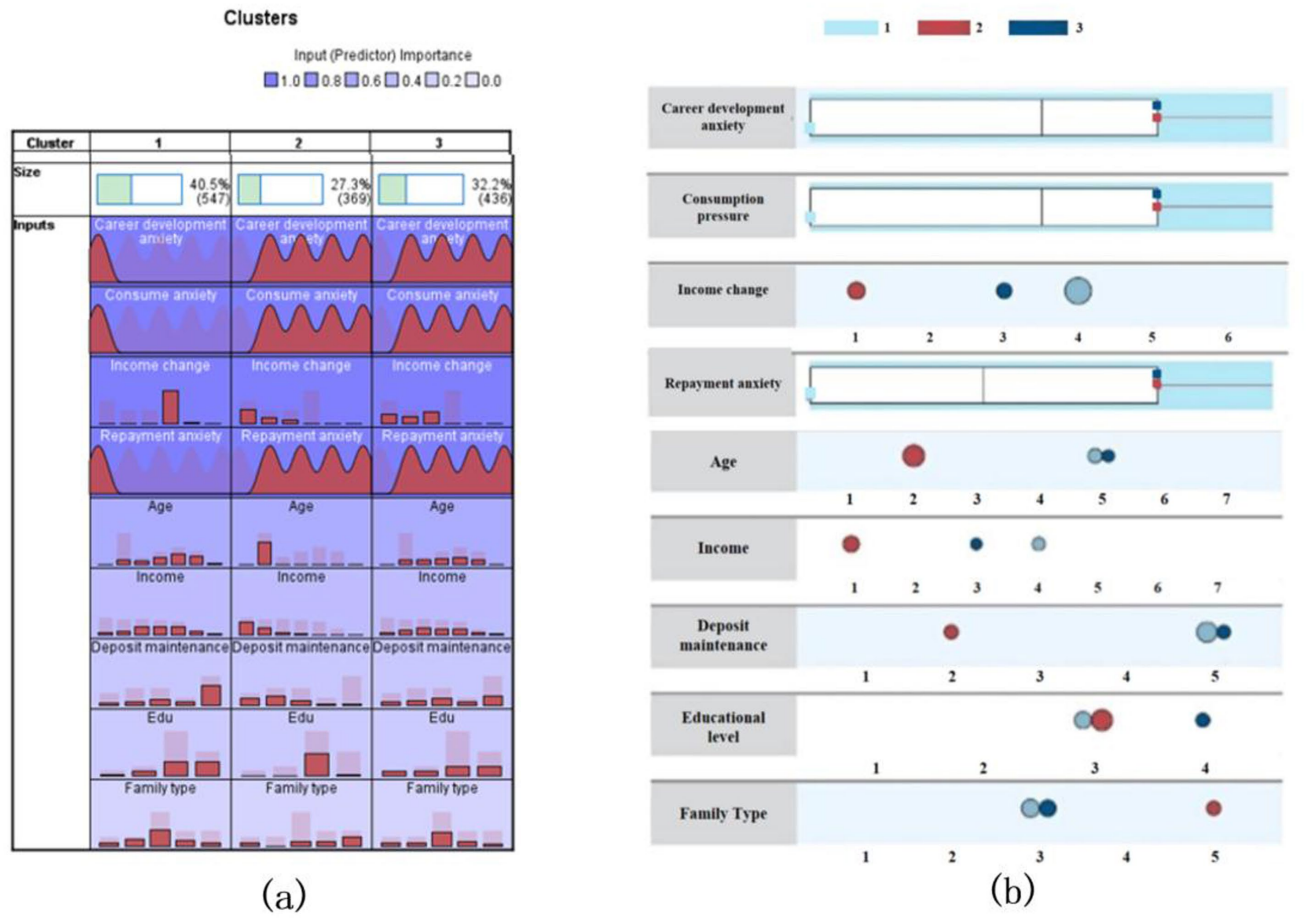
Clustering results.

Figure 2a reveals three distinct sample classifications with balanced proportions: Type 1 (40.5%), Type 2 (27.3%), and Type 3 (32.2%). Nine input variables were ranked by their classification importance, with career development anxiety (1st), consumption anxiety (2nd), and income change (3rd) emerging as the most influential factors. While career and consumption anxiety exhibited similar frequency distributions across groups, repayment anxiety (4th) had a notably weaker impact, particularly in Type 3. Income change showed marked divergence: Type 1 residents experienced income increases during the pandemic, whereas Types 2 and 3 faced declines and Type 2 suffered the steepest drops. Deposit maintenance had minimal influence.

Demographically, age was the most critical individual difference: Type 2 predominantly comprised younger adults (18–25 years), contrasting with the balanced age distribution in Types 1 and 3. Type 2 further differed in socioeconomic traits: low monthly family income, undergraduate education dominance, and unmarried status (e.g., living alone or with parents). In contrast, Types 1 and 3 were middle-to-high-income earners (with Type 3 having more high-income individuals) and shared similar education levels and family structures.

Age was the most important variable in terms of individual differences. The first and the third sample types showed a relatively balanced distribution for each age group, while the second sample type had a prominent distribution for the second age group (18–25 years). A similar prominent distribution was also found for the rest of the individual differences. The second type of sample residents’ monthly family income comprised of respondents who earned low incomes, who had a largely undergraduate level education, and whose family type mostly included those who were unmarried (e.g., living alone or with parents). Therefore, career and consumption anxieties as well as income volatility drive classification, with Type 2 representing a vulnerable cohort of younger, low-income individuals requiring targeted policy interventions to address pandemic-induced financial strain.

Our classification framework identifies two primary drivers: income decline anxiety and actual income change, supplemented by demographic factors (age, marital status) and deposit stability as secondary moderators (Figure 2b). Three distinct cohorts emerged: Type 1 (middle-aged, high-income married families with stable/growing income and minimal anxiety), Type 2 (unmarried youth aged 18–25 with low-middle incomes and severe income declines, facing acute financial strain), and Type 3 (middle-aged, middle-income married families experiencing mild declines, cushioned by savings). These patterns underscore how economic shocks interact with demographic resilience, emphasizing the need for targeted policies to support vulnerable groups like Type 2, whose financial fragility risks long-term socioeconomic consequences.

### 3.3 Multiple response analysis

**Figure 3:**
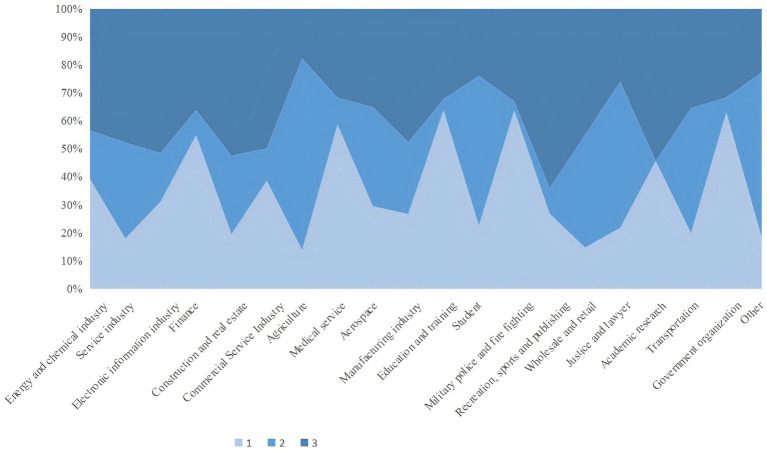
Distribution of family members’ occupation types in cluster samples.

**Figure 4:**
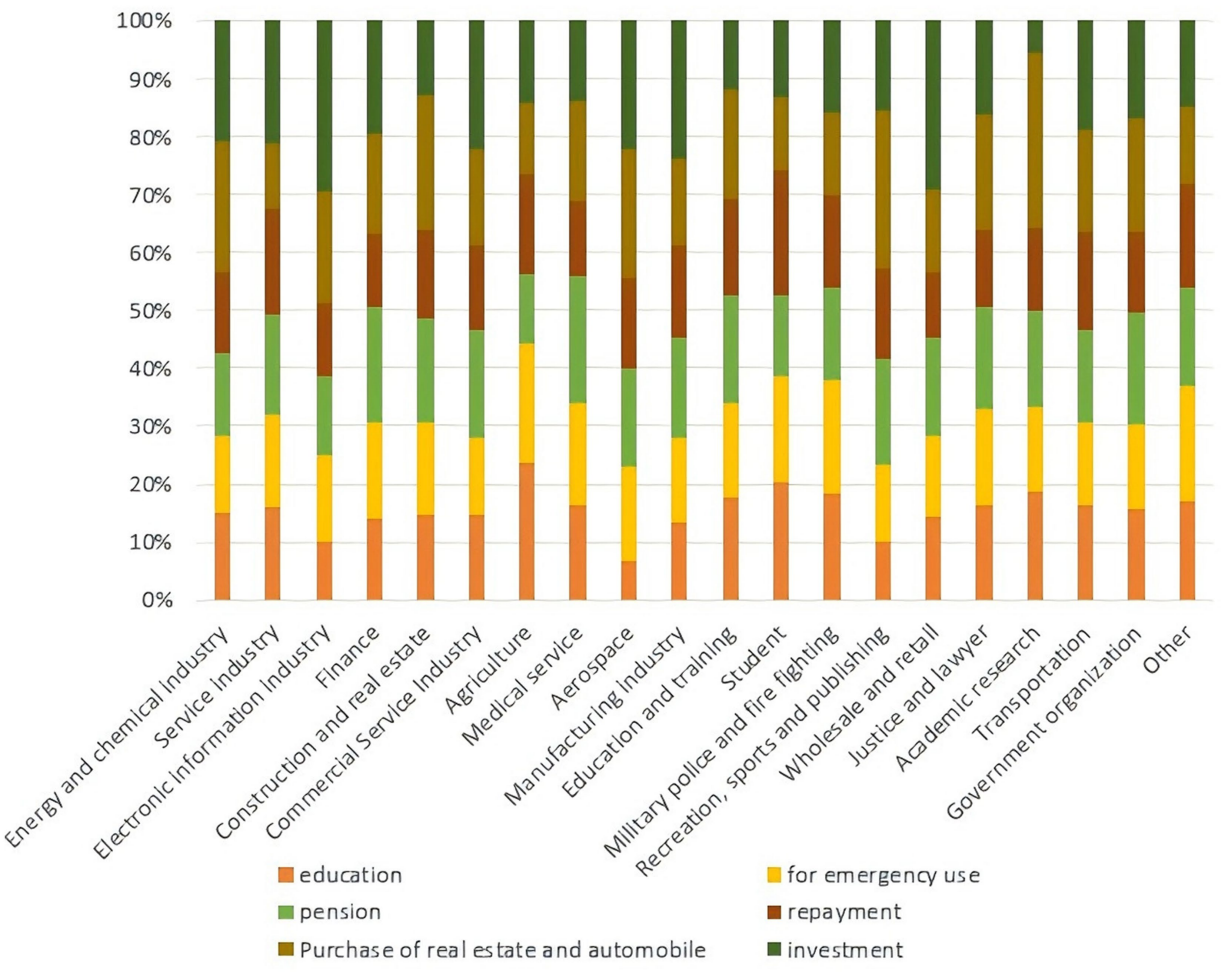
The relationship between deposit motives and the family members’ occupation types.

In this questionnaire, family members’ occupational types and deposit motives are associated with multiple topics. In order to further explore the distribution of family members’ occupational types among the three categories of samples, and to examine the impact of the epidemic on the industrial economy and family economy, as well as the relationship between family deposit motives, industry and behavior selection, we carried out a multiple response analysis of the clustering results, family members’ occupational types, and deposit motives. To improve the analyzability of the results, we classified 50 types of occupations in the questionnaire into 20 categories. The results of the analysis of the responses are shown in Figures 3 and 4.

#### 3.3.1 The correlation between family occupational types and the clustering results

Figure 3 illustrates the occupational distribution across the three sample categories, aligning with their income stability and pandemic vulnerability. Type 1 families predominantly worked in stable sectors (energy, healthcare, education, government) with fixed incomes minimally disrupted by the pandemic. In contrast, Type 2 were concentrated in market-dependent industries (finance, manufacturing, retail, agriculture), which faced severe COVID-19 impacts. Type 3 mirrored Type 2’s occupational profile but with notable shifts: higher proportions in lucrative sectors (construction, aerospace, academia) and reduced representation in agriculture and student populations.

This occupational stratification correlates with the clusters’ “baseline income” and “post-pandemic income changes” observed earlier: stable industries shielded Type 1 from financial shocks, while Type 2’s market-reliant occupations exacerbated their vulnerability. Type 3’s partial transition to higher-income sectors likely buffered moderate income declines, reflecting adaptive resilience. These patterns underscore how occupational structures mediate economic resilience during crises, informing targeted sectoral interventions to mitigate pandemic-induced disparities.

#### 3.3.2 The correlation between reasons for saving money and the clustering results

Analysis of saving motives across the three clusters revealed education investment (self/children) as the top priority, followed by emergency preparedness, consistent across all groups. Notably, Cluster 2 characterized by younger, low-middle-income individuals, allocated significantly fewer resources to elderly care, real estate, automobiles, and investments compared to Clusters 1 and 3. While Clusters 1 (high-income) and 3 (middle-income) showed comparable savings proportions for property and vehicles, Cluster 3 prioritized investments and loan repayments more heavily.

These patterns align with occupational disparities: Cluster 1’s stable industries (e.g., healthcare, government) enabled long-term asset accumulation, whereas Cluster 2’s market-dependent sectors (retail, agriculture) limited surplus funds for non-essential goals. Cluster 3’s partially transitioning to higher-income sectors like aerospace or supported moderate investment capacity despite pandemic-induced income declines (Figure 4). The findings underscore how occupational stability mediates financial resilience, with Cluster 2’s limited savings diversification heightening vulnerability to crises. Targeted financial literacy programs and flexible saving instruments are critical to address this cohort’s compounded risks.

### 3.4 Conditional process model test

Based on the above analysis, we summarized the relative characteristics of the three categories of samples (Table 3). Income declining anxiety perception (IDAP) and Income change (IC) were taken as the primary indicators, while the original income level was used as a secondary indicator.

**Table 3:** Overview of the main characteristics of the three clusters.

Cluster	IDAP	IC	Income	IDSC	Industry	DMA	DMO
1	×	×	Upper-middle	High	Stable	Long	Balanced
2	√	√	Low	Low	Market dependence	Short	Conservative
3	√	√	Middle	Low	Market dependence	Long	Risk

Compared with the first category of samples, the other two categories experienced anxiety about declining income; the original income of respondents in the third category was similar to that of respondents in the first category, whereas those in the second category earned the lowest income. Therefore, we defined the first category of samples as “high-income glide impact cognitive level (HIDSC)” residents (i.e., the experience perception of an income glide and income glide impact anxiety was far away, and the cognitive solution was more abstract); the second and third categories included “low-income glide impact cognitive level (LIDSC)” residents (i.e., personally experience income glide and income glide anxiety perception, and the cognitive solution was more concrete). The Industry, Deposits maintenance (DMA), and the Deposit motives (DMO) were the background indicators of behavioral selection, which were the secondary entry points for the discussion.

In order to explore whether the perceived anxiety of residents with different IDSC could mediate their subsequent savings and consumption behavior, we transformed three samples into dummy variables. The first category was set as reference item X0, and the other two items were set as X1 and X2, which formed the independent variable input model; Living supplies shortage anxiety perception (LA) and the Prevention and control supplies shortage anxiety perception (PA) were the intermediary variable, and low risk saving behavior (LRB) and advanced consumption behavior (ACB) were the dependent variables. Considering the impact of the epidemic, the national level of medical trust, media trust and the community trust were integrated into the model as a moderating variable to provide a situational interpretation for the model.

In order to address a possible regulatory effect, we used Process Macro (Model 59) in SPSS to test the proposed research hypothesis. After substituting the variables in this study into Model 59. The results of the conditional process model test are shown in Tables 4, 5.

**Table 4:** Test results of conditional process model when ACB as dependent variable.

Variables	Model 5 (DV:LA)				Model 6 (DV:ACB)					Model 7 (DV:PA)				Model 8 (DV:ACB)			
	coeff	se	*t*	*p*	coeff	se	*t*	*p*		coeff	se	*t*	*p*	coeff	se	*t*	*p*
Constant	−0.105	0.042	−2.523	0.012	−0.129	0.038	−3.377	0.001	Constant	−0.163	0.041	−3.062	0.002	−0.122	0.038	−3.225	0.001
X1	0.149	0.068	2.202	0.028	0.250	0.062	4.063	0.000	X1	0.193	0.067	2.875	0.004	0.240	0.061	3.943	0.000
X2	0.196	0.063	3.105	0.002	0.109	0.058	1.894	0.059	X2	0.242	0.063	3.862	0.000	0.089	0.057	1.560	0.119
ST	0.159	0.043	3.722	0.000	0.127	0.039	3.228	0.001	ST	0.204	0.042	4.816	0.000	0.108	0.039	2.757	0.006
X1* ST	0.085	0.067	1.271	0.204	0.074	0.061	1.208	0.227	X1* ST	0.018	0.066	0.264	0.792	0.088	0.061	1.446	0.148
X2* ST	0.104	0.064	1.625	0.105	0.092	0.058	1.578	0.115	X2* ST	0.093	0.064	1.470	0.142	0.089	0.057	1.542	0.123
LA					0.338	0.025	13.487	0.000	PA					0.363	0.025	14.284	0.000
LA* ST					0.111	0.022	5.072	0.000	PA* ST					0.106	0.022	4.724	0.000
*R*^2^	0.057				0.223				*R*^2^	0.071				0.243			
*F*	16.266^***^				55.000^***^				*F*	20.508^***^				61.500^***^			

DV, Dependent Variable.

**Table 5:** Significant critical points of J–N test.

Model	Value	%below	% above	Effect	se	*t*	*p*	LLCI	ULCI
LA-LRB	1.3362	0.5917	99.4083	0.18	0.0918	1.9617	0.05	0	0.3601
LA-ACB	2.5692	2.5888	97.4112	0.1084	0.0553	1.9617	0.05	0	0.2168
PA-LRB	1.921	0.8136	99.1864	0.159	0.081	1.9617	0.05	0	0.3179
PA-ACB	2.4099	1.8491	98.1509	0.1236	0.063	1.9617	0.05	0	0.2471

From Table 6, we can see that in Model 1 and Model 3, the influence of independent variables X1 and X2 on LA or PA was positive and significant. However, the *p* value of the interaction term between the independent variable and the regulating variable was more than 0.05, and path 4 was not significant. When LRB was a dependent variable (Model 2), the *p* value of the interaction term between the independent variable and the moderating variable still did not meet the significance condition, so path 5 did not hold. That is to say, the impact of IDSC was not the key to determining the significant adjustment path 1 and 2 of social trust.

**Table 6:** Test results of conditional process model when LRB as dependent variable.

Variables	Model 1 (DV:LA)				Model 2 (DV:LRB)					Model 3 (DV:PA)				Model 4 (DV:LRB)			
	coeff	se	*t*	*p*	coeff	se	*t*	*p*		coeff	se	*t*	*p*	coeff	se	*t*	*p*
Constant	−0.105	0.042	−2.523	0.012	−0.154	0.038	−4.086	0.000	Constant	−0.127	0.041	−3.062	0.002	−0.155	0.039	−4.026	0.000
X1	0.149	0.068	2.202	0.028	0.287	0.061	4.711	0.000	X1	0.193	0.067	2.875	0.004	0.284	0.062	4.584	0.000
X2	0.196	0.063	3.105	0.002	0.210	0.057	3.697	0.000	X2	0.242	0.063	3.862	0.000	0.205	0.058	3.543	0.000
ST	0.159	0.043	3.722	0.000	0.220	0.039	5.678	0.000	ST	0.204	0.042	4.816	0.000	0.216	0.040	5.431	0.000
X1* ST	0.085	0.067	1.271	0.204	0.009	0.060	0.155	0.877	X1* ST	0.018	0.066	0.264	0.792	0.027	0.062	0.433	0.665
X2* ST	0.104	0.064	1.625	0.105	0.043	0.058	0.741	0.459	X2* ST	0.093	0.064	1.470	0.142	0.048	0.058	0.822	0.411
LA					0.338	0.025	13.650	0.000	PA					0.338	0.025	13.650	0.000
LA* ST					0.044	0.022	2.033	0.042	PA* ST					0.049	0.023	2.166	0.031
*R*^2^	0.057				0.241				*R*^2^	0.071				0.218			
*F*	16.266^***^				60.969^***^				*F*	20.508 ^***^				53.483^***^			

DV, Dependent Variable.

However, when the intermediary variables were included in the model, *R*^2^ was enhanced, and the explanatory power of the model improved. The coefficients of LA * ST and PA * ST were both positive and significant. Therefore, when LRB was the dependent variable, social trust adjusted path 3 through to path 6. This meant that the residents (X1, X2) of LIDSC were more likely to be positively regulated by social trust and undertake LRB after mediating material PA. Moreover, the interaction between material PA and social trust had a stronger impact on residents of LIDSC. However, the interaction between X1 and X2 and social trust had no significant impact on LRB, i.e., social trust did not constitute a moderator in the direct impact path of LIDSC residents on LRB.

Comparable to LRB, when ACB was the dependent variable (Table 4), the influence of X1 and X2 on LA or PA was positive and significant, but the regulatory effect of social trust on paths 1 and 2 was not significant. This indicated that IDSC could not play a significant role in the first half of the conditional process model. After the mediating effect of material PA, the regulatory effect of ACB received by residents of LIDSC was about 0.1 times that of residents of HIDSC (X0); among them, the regulatory effect of social trust was relatively stronger with respect to the influence of material PA on ACB. Social trust also did not moderate the direct path of LIDSC residents to ACB.

### 3.5 Conditional effect

#### 3.5.1 Conditional indirect effect

A bootstrap test was then conducted for the indirect effect of the condition, and we substituted the adjustment variables into M − 1SD, m, and M + 1SD values, respectively, and the result is presented in Appendix 2. We judged whether the intermediary exists according to whether the interval value of BootLLCI contained 0. The results of the model with LRB as the dependent variable showed that the mediating effect was valid only when the independent variable was X2 and the mediating variable was PA. In other words, compared with HIDCS, the mediating effect of LA was observed among residents (X1, X2) of LIDSC only when the moderating variable was greater than or equal to the mean value, regardless of income. In respect to the intermediary variable “prevention and control of material AP,” residents with relatively high original incomes in LIDSC (X2), and regardless of the size of the adjustment variable, always played an intermediary role in LRB. That is to say, the lower the level of social trust, the smaller the mediating effect of PA in regard to the behavioral choice made by residents of LIDSC and HIDCS.

The indirect effect test of the bootstrap condition with ACB as the dependent variable was similar to that of LRB: When the adjustment variable was M + 1SD, the impact of an IDSC was more significant. When the adjustment variable was greater than or equal to the average value, the mediating effect increased with the increase in the adjustment intensity: The mediating effect of residents’ AP of LIDSC was about 0.05–0.1 times that of residents of HIDSC. The mediating effect of PA among residents with higher original incomes was the largest, i.e., 0.134 times of X0. Compared with HIDSC, the impact of LIDSC played a more indirect role in behavioral selection by means of the intermediary role of PA.

By comparing the mediating variables horizontally, we can see that the indirect mediating effect of PA was positively related to the moderating effect of social trust. Except for the mediating coefficient when the moderating variable of X1 was M + 1SD, the other results showed that the mediating effect of PA was better under the same moderating intensity. This meant that compared with HIDSC, LIDSC residents were more sensitive to the mediating role of AP; and in most cases, the mediating role of PA was more prominent. However, when the lower income group (X1) of LIDSC residents had a higher level of trust in the society, the mediating effect of LA on the behavioral choice reversed the mediating effect of prevention and control supplies. This showed that after controlling for other factors, the moderating effect of high social trust diminished the pessimistic attitude of low income residents who had a high level of anxiety about declining income as a result of the epidemic, and they focused on the demand for daily living supplies.

When there was a medium level of social trust (mean value), the mediating intensity of X1 residents’ LA was greater in LRB. Other effective indirect paths showed that when the level of social trust was higher than the average, the demands of ACB among residents of LIDSC for mediating ASS was greater. In the context of the epidemic, compared with residents of LIDSC, a higher level of social trust moderation and a higher original income were key factors for residents to engage in ACB. However, the behavioral choice of X2 residents was influenced by the more mediating effect of AP, and ACB will have a greater mediating effect. This showed that residents of LIDSC who had higher original incomes were more sensitive to the development of the epidemic and to the changes to daily life during the epidemic. If the mediating effect of AP was strong, residents paid greater attention to planning issues related to family property and they were more likely to choose ACB.

In summary, the interval value of BootLLCI contains 0, which indicated that the mediating effect of AP varied with the moderating variables. This meant that there was a moderating mediating effect, and the conditional process model was established. The residents of LIDSC will have a greater AP and experience more negative emotions because of their concerns about the quantity and quality of supplies during the epidemic. As a result, the impact of declining income on the level of the cognitive solution, through the intermediary variable of AP, had a stronger indirect effect on the behavioral selection. On the whole, the awareness of PA played a more intermediary role; when the LIDSC group made behavioral choices, residents with higher incomes were more affected by the fluctuation in supply conditions during the epidemic, especially when they decided to make decisions about ACB.

#### 3.5.2 Conditional direct effect

After controlling the intermediary effect of AP, the conditional direct effect in the model was tested and discussed. The results of the conditional direct effect of the bootstrap test are summarized in Appendix 3.

On the whole, compared with HIDSC, LIDSC had a stronger direct effect on HSCB, and groups with higher levels of social trust had a stronger direct effect. When the dependent variable was LRB, the direct effects of the three levels of social trust moderation were significant (the bootstrap confidence interval did not contain 0). In the LIDSC group, the residents with lower incomes had a stronger direct impact on the behavioral choice. This showed that IDSC led to a significant difference in the direct effect on the choice of LRB, and the impact of declining incomes among residents with lower original incomes played a stronger role. That is to say, the LIDSC group showed a preference for LRB due to their perception of the impact that would result from income changes, and the LRB of individuals with lower original income levels was more strongly influenced by perceived income declines.

When the dependent variable was ACB, the direct effect path was not significant when the level of social trust was low. The direct impact of declining income on ACB was only observed when the level of social trust was M + 1SD. This indicated that for groups that have low levels of social trust, the impact of declining income had no significant difference on whether residents will engage in ACB in the future. However, when the level of social trust was higher, the impact of low income decline on the understanding level will play a more direct role in ACB; Similar to LRB, compared with high income samples, LIDSC played a more important role when low income samples choose ACB.

Comparing the direct effects of these two behavior choices, we found that when the level of social trust was less than or equal to the average, LRB was more directly affected. When the level of social trust level was higher than the average, the LIDSC group with lower original incomes had a slightly stronger direct effect on ACB. For the group with higher original incomes, the direct effect corresponding to ACB was significantly weaker than that of LRB. This showed that, compared with HIDSC, LIDSC preferred LRB when they chose two behaviors; only a high level of social trust could alleviate the anxiety caused by the impact perception to a certain extent, and cause residents to show a preference for ACB.

Compared with the high income sample, the low income sample in the LIDSC group was more likely to undertake LRB and ACB. Combined with our analysis of the motives for which participants saved money, this may also be related to the preference observed among X2 (higher income) groups who wished to save money for investment motives. Therefore, we believe that the choice of behavior was not only related to the perception of the dynamic impact of declining income, but it was also affected by the original income level and previous saving habits.

From the perspective of the direct effect and the growth trend of the social trust level, when the social trust level increased, the impact of declining income on the perception of the direct effect of LRB increased more slowly. This indicated that LRB was a basic behavioral choice, and it did not require too much regulation in the area of social trust, especially among low income residents with LIDSC. However, when engaging in ACB, in addition to the impact of perceptions of declining income, it was more dependent on social trust. Nevertheless, in contrast with the assumption, a high level of social trust did not encourage residents to plan property-related matters in the long-term, which was particularly obvious in the case of low-income residents with LIDSC. Perhaps due to their higher level of trust in society, residents with LIDSC hoped to enjoy vested interests at this stage, because there will be more uncertainty in the long-term future.

In summary, compared with the HIDSC, the impact of IDSC plays a more direct role when LIDSC making LRB and ACB. Furthermore, the higher the social trust level of low-income groups, the low level of IDSC plays a greater role in residents’ savings and consumption behavior.

### 3.6 Johnson–Neyman slope test and hypothesis testing

The Johnson–Neyman (J–N) slope test can determine the significant critical point of the adjustment term at the significant edge, which allowed us to define the interval value of the adjustment variable when the simple slope is significant (not 0). We tested the J–N simple slope of the significant adjustment path. The significant critical point information of the four models is summarized in Table 5.

**Figure 5:**
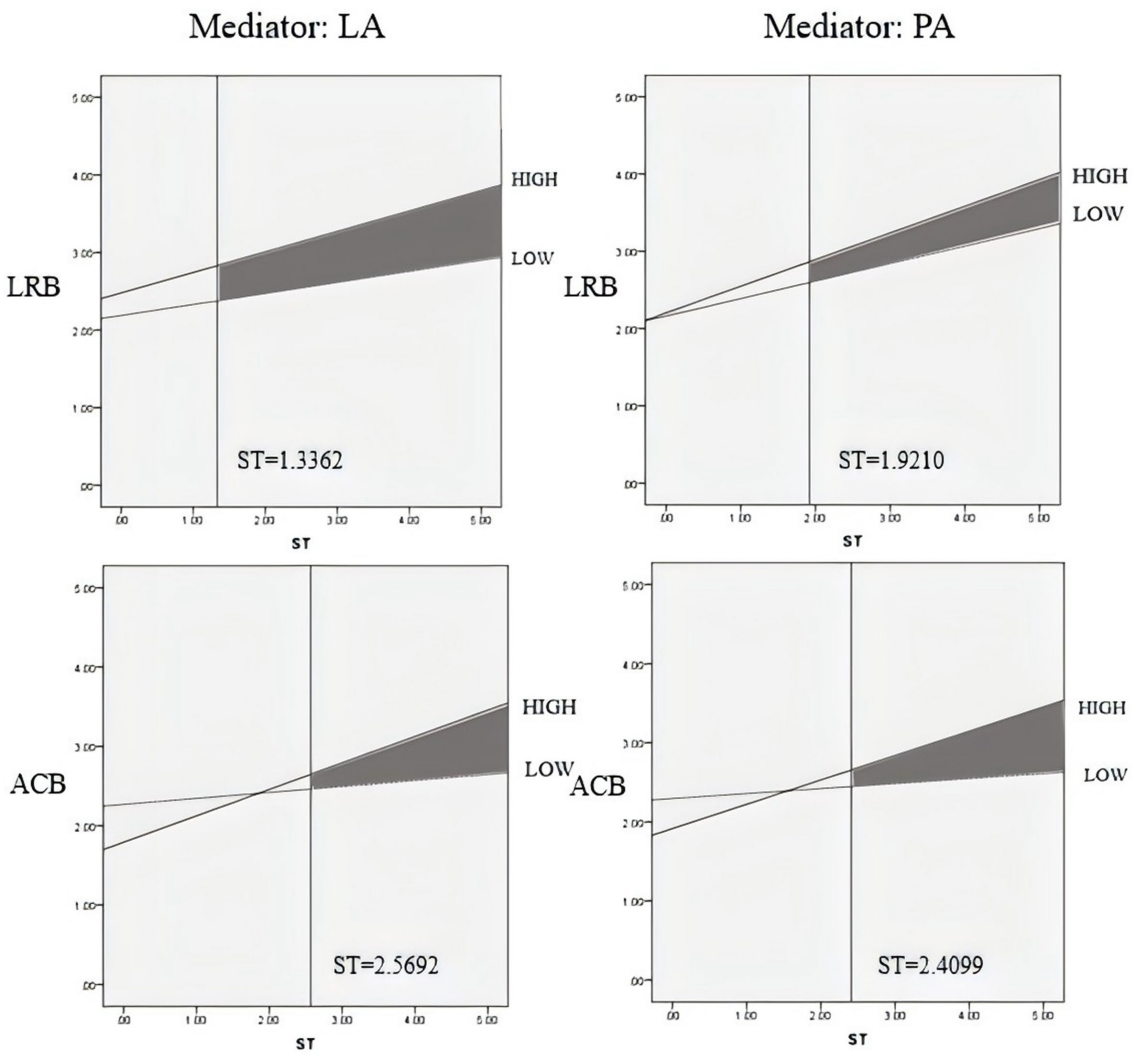
M ± 1SD & J–N mixed slope diagram.

Figure 5 presents slope test results (M ± 1SD) examining social trust’s moderation on behavioral choices. When social trust exceeded the significant threshold, independent variables in all four models showed steeper positive slopes for both LRB and ACB, indicating that LIDSC residents with high material anxiety (PA) were more likely to adopt these behaviors under elevated trust. Conversely, low social trust weakened PA’s impact, particularly for ACB models, which demanded higher trust levels to trigger timely, enjoyment-oriented decisions. Notably, social trust minimally influenced LRB/ACB among residents with low PA. Comparative analysis revealed weaker mediating effects for latent anxiety (LA) versus PA.

Transitioning from LRB to ACB amplified social trust’s moderating demands, while PA’s robust mediation reduced disparities between HIDSC and LIDSC residents in LRB choices. The similarity of trust thresholds across PA-mediated models further underscored its dominant mediating role. These patterns align with earlier discussions, partially validating Hypothesis 3 by demonstrating that social trust primarily moderates outcomes indirectly through PA rather than direct pathways. The findings highlight context-dependent trust efficacy: while high trust buffers material anxiety’s behavioral impacts, systemic inequities persist, as LIDSC populations require disproportionately higher trust levels to achieve comparable resilience.

## 4 Discussion

Based on the measurement of income status, psychological status, and future savings and consumption behavior during the epidemic period as well as the verification of the conditional process model, the results showed that residents with LIDSC were more likely to prefer low risk savings behavior and short-time consumption behavior. This result verified and supported the conclusion that “people with a high level of understanding are more willing to take risks or wait,” from the perspective of experience distance (Abraham et al., 2023; Chen and He, 2011). This showed that, as a group whose income did not decline significantly due to the epidemic, “income decline” did not play a significant role, and the psychological distance was high. Therefore, such groups were more likely to choose high risk savings behavior, which was not feasible but attractive (Liberman and Trope, 1998), as well as advance consumption behavior, which was related to the increase in the value weight over time, which emphasis the importance of subjective judgment of future time in intertemporal preferences (Kim et al., 2012; Trope and Liberman, 2010).

However, most existing research studies focused on the main effect between the cognitive level and the behavioral choice, and research on the mechanism of the mediating and moderating action outside the main effect was not sufficient (Figner and Weber, 2011; Xu et al., 2024b). From the perspective of the 2019 novel coronavirus epidemic, the intermediary variable was “anxiety about supply shortages” as a result of the epidemic, and social trust was the decision-making situational variable. The results revealed that when there was a low level of social trust, there was no significant difference in the mediating role of supply shortages anxiety between the two types of residents. When the level of social trust was above average, the residents of LIDSC were more sensitive to material anxiety. That is to say, anxiety, as a negative emotion, usually caused people to overestimate risks (Lerner and Keltner, 2001; Immordino et al., 2021) and show a more thoughtful financial decisions such as conservative low risk savings behavior (Talwar et al., 2021). Moreover, individuals with negative emotions exhibit reduced patience for delayed income due to steepening temporal discounting and a preference for immediate gratification, driven by diminished cognitive control and heightened impulsivity (Ifcher and Zarghamee, 2011; Lei et al., 2024). The higher income group in LIDSC showed a stronger tendency to choose ACB once material anxiety perception began to play an intermediary role. This finding is consistent with the conclusions reached by Zaman et al. (2012): When there is a scarcity of materials, low income groups as well as higher income groups are driven by the current demand, and they are more inclined to relinquish long-term interests because of the acquired value.

Social trust only effectively moderates the action path of material anxiety perception on behavioral choice, and it has a positive regulatory effect. When there is a higher level of social trust, residents with higher levels of anxiety about supply shortages will choose low risk savings consumption behavior and advanced consumption behavior. Contrary to our hypothesis, social trust did not alleviate perceived anxiety about supply shortages, which caused LIDSC residents to consider choosing long-term savings consumption behavior. However, when we analyzed the current level of social trust among the sample, we found that the level of social trust among LIDSC residents was generally higher than that of HIDSC residents. This showed that although the income of residents in LIDSC declined, and they sensed the impending impact of this income decline, and they faced heightened supply shortage anxiety, primarily stemming from COVID-19 pandemic concerns and its socioeconomic repercussions, such as disrupted supply chains and income instability, which amplified perceived scarcity risks (Adams-Prassl et al., 2020; Hristov et al., 2022). Nevertheless, at this stage, the residents had full trust in medical treatments, media publicity, and the ability of the government and the community to govern. In other words, they were more satisfied with the current social situation and they preferred to discount at the present time; while HIDSC residents had more positive expectations about the future, their delayed discount rate was relatively low (Donnell et al., 2017), so they had a stronger “effective desire to accumulate.”

In addition, apart from the level of the income decline, the individual differences in the sample also had influenced the choice of behavior. From the perspective of income levels and occupation types, the occupations of the second household type mostly included farmers and those working in service industries, except students, while the first and third types of samples primarily included middle-class residents with relatively stable occupations. Hendon et al. (1998) and Alexander (2022) pointed out that the middle class often engages in a more rational and planned form of behavior, an idea which is supported by the findings of present study. The first sample type was more inclined to choose a longer delay and risk options in regard to the cross-period behavior; the second sample type, which included the group with a relatively low level of saving maintenance and stratum, paid less attention to long-term results; the third sample type was more inclined to choose low risk saving compared with the first sample of residents with a low level of awareness in respect to declining income. However, the savings and consumption behavior before the epidemic was similar to that observed among the first sample of residents. Therefore, even though savings and consumption behavior changed, the preference for this behavior was still weaker than that observed among the second sample of residents because of behavioral inertia.

In summary, the samples X1 and X2 (i.e., low income decline awareness level) preferred low risk savings and advanced consumption behavior. Therefore, Hypothesis 1 was verified. The mediating role of anxiety about perceived supply shortages between LIDSC and the two behaviors was established, i.e., LIDSC residents were more sensitive to the mediating role of anxiety about perceived supply shortages when they engaged in low risk savings and advanced consumption behaviors. Hypothesis 2 was verified. However, social trust did not play a role in adjusting the direct path between LIDSC residents and supplies shortage anxiety. Hypothesis 3(a) was rejected. Moreover, according to the bootstrap test, social trust moderated the direct path between LIDSC and HSCB, and the higher level of social trust among low income groups, the greater the role played by LIDSC in respect to decision-making and behaviors. Hypothesis 3(b) was verified. Social trust played a significant role in the terms of how supplies shortage anxiety may influence the two behavioral choices, which meant that, in comparison with residents with a high income awareness level, social trust can more effectively regulate the material anxiety perception of LIDSC residents’ choice regarding low risk savings and advanced consumption behavior. Hypothesis 3(c) was verified.

Above all, compared with residents who had a stable income during the epidemic, residents with declining incomes were more likely to engage in low risk savings behavior and advanced consumption behavior in the future. This conclusion verified the Construal-level theory. On this basis, we also found that residents with LIDSC had a stronger preference for low income groups, higher income groups were more sensitive to anxiety about perceived supply shortages during the epidemic, as well as the positive regulatory role of social trust. This study can provide a micro-level perspective and reference for the prediction of individuals’ behavioral choices after similar events.

The study’s sample, shaped by the logistical constraints of data collection during the pandemic (e.g., reliance on online survey platforms that disproportionately attract educated urban populations) predominantly comprised urban residents with higher education levels and income, and the respondents to the questionnaire are mostly young and middle-aged residents who can skillfully operate electronic equipment, which may limit the generalizability of findings to rural or less-educated or aging populations. Future research should prioritize broader demographic representation to validate these results. In addition, the study’s temporal scope focusing on short-term adjustments within 1 year post-shock, precludes insights into long-term behavioral dynamics. For instance, whether initial risk aversion gives way to fatalism or innovation over time remains untested. Future work should integrate longitudinal tracking with policy research to map adaptive trajectories across economic cycles.

## Data Availability

The raw data supporting the conclusions of this article will be made available by the authors without undue reservation.

## References

[ref1] Abeysinghe S. Leppold C. Ozaki A. Morita M. Tsubokura M. (2017). Disappearing everyday materials: the displacement of medical resources following disaster in Fukushima, Japan. Soc. Sci. Med. 191, 117–124. doi: 10.1016/j.socscimed.2017.09.011, PMID: 28917620 PMC5630202

[ref2] Abraham D. Powers J. P. Mcrae K. (2023). Emotional appraisal, psychological distance and construal level: implications for cognitive reappraisal. Emot. Rev. 15, 313–331. doi: 10.1177/17540739231196577

[ref3] Adams-Prassl A. Boneva T. Golin M. Rauh C. (2020). Inequality in the impact of the coronavirus shock: evidence from real time surveys. J. Public Econ. 189:104245. doi: 10.1016/j.jpubeco.2020.104245

[ref4] Al-Dahash H. Kulatunga U. Thayaparan M. (2019). Weaknesses during the disaster response management resulting from war operations and terrorism in Iraq. Int. J. Disaster Risk Reduct. 34, 295–304. doi: 10.1016/j.ijdrr.2018.12.003

[ref5] Alexander C. (2022). The simple bare necessities: scales and paradoxes of thrift on a London public housing estate. Comp. Stud. Soc. Hist. 64, 934–965. doi: 10.1017/S0010417522000159

[ref6] Bchir M. Willinger M. (2013). Does a membership fee foster successful public good provision? An experimental investigation of the provision of a step-level collective good, Public Choice, 157, 25–39. doi: 10.1007/s11127-012-9929-9

[ref7] Brügger A. Morton T. A. Dessai S. (2016). “Proximising” climate change reconsidered: a construal level theory perspective. J. Environ. Psychol. 46, 125–142. doi: 10.1016/j.jenvp.2016.04.004

[ref8] Cassar A. Healy A. Von Kessler C. (2017). Trust, risk, and time preferences after a natural disaster: experimental evidence from Thailand. World Dev. 94, 90–105. doi: 10.1016/j.worlddev.2016.12.042

[ref9] Chen H. He G. (2011). The influence of construal level on intertemporal choice and risk choice. Acta Psychol. Sin. 43, 442–452. doi: 10.3724/SP.J.1041.2011.00442

[ref10] Daniali H. Martinussen M. Flaten M. A. (2023). A global meta-analysis of depression, anxiety, and stress before and during COVID-19. Health Psychol. 42, 124–138. doi: 10.1037/hea0001259, PMID: 36802363

[ref11] Dhar R. Kim E. (2007). Seeing the forest or the trees: implications of construal level theory for consumer choice. J. Consum. Psychol. 17, 96–100. doi: 10.1016/S1057-7408(07)70014-1

[ref12] Donnell S. Daniel T. O. Epstein L. H. (2017). Does goal relevant episodic future thinking amplify the effect on delay discounting? Conscious. Cogn. 57, 10–16. doi: 10.1016/j.concog.2017.02.014 PMC5651988 28282631

[ref13] Evans D. R. Yemeke T. T. Kiracho E. E. Mutebi A. Ozawa S. (2019). Trust in vaccines and medicines in Uganda. Vaccine 37, 6008–6015. doi: 10.1016/j.vaccine.2019.07.022, PMID: 31447127

[ref14] Fan X. C. Lu J. Y. Qiu M. X. Xiao X. (2023). Changes in travel behaviors and intentions during the COVID-19 pandemic and recovery period: a case study of China. J. Outdoor Recreat. Tourism 41:100522. doi: 10.1016/j.jort.2022.100522, PMID: 37521263 PMC9046066

[ref15] Fiedler K. Jung J. Wänke M. Alexopoulos T. (2012). On the relations between distinct aspects of psychological distance: an ecological basis of construal-level theory. J. Exp. Soc. Psychol. 48, 1014–1025. doi: 10.1016/j.jesp.2012.03.013

[ref16] Figner B. Weber E. U. (2011). Who takes risks when and why? Determinants of risk taking. Curr. Dir. Psychol. Sci. 20, 211–216. doi: 10.1177/0963721411415790

[ref17] Frederick S. Loewenstein G. O'Donoghue T. (2002). Time discounting and time preference: a critical review. J. Eco. Lit. 2, 351–401. doi: 10.1257/002205102320161311

[ref18] Geirdal A. O. Ruffolo M. Leung J. Thygesen H. Price D. Bonsaksen T.. (2021). Mental health, quality of life, wellbeing, loneliness and use of social media in a time of social distancing during the COVID-19 outbreak. A cross-country comparative study. J. Ment. Health 30, 148–155. doi: 10.1080/09638237.2021.1875413 33689546

[ref19] Gilmore B. Ndejjo R. Tchetchia A. de Claro V. Nyamupachitu-Mago E. Diallo A. A.. (2020). Community engagement for COVID-19 prevention and control: a rapid evidence synthesis. BMJ Glob. Health 5:e003188. doi: 10.1136/bmjgh-2020-003188 PMC7554411 33051285

[ref20] Green L. Myerson J. (1996). Exponential versus hyperbolic discounting of delayed outcomes: risk and waiting time. Am. Zool. 36, 496–505. doi: 10.1093/icb/36.4.496

[ref21] Hendon D. W. Williams E. L. Huffman D. E. (1998). Social class system revisited. J. Bus. Res. 3, 259–270.

[ref22] Hristov H. Millard J. Pravst I. Janssen M. (2022). European household spending and socio-economic impacts on food behavior during the first wave of COVID-19. Front. Nutr. 9:869101. doi: 10.3389/fnut.2022.869091, PMID: 35990318 PMC9382126

[ref23] Ifcher J. Zarghamee H. (2011). Positive affect and overconfidence: a laboratory investigation. J. Neur. Psych. Econ. 3, 15–150. doi: 10.1037/npe0000022

[ref24] Immordino G. Jappelli T. Oliviero T. Zazzaro A. (2021). Fear of covid-19 contagion and consumption: evidence from a survey of Italian households. Health Econ. 31, 496–507. doi: 10.1002/hec.4464, PMID: 34962332 PMC9015365

[ref25] Janssen M. Chang B. P. Hristov H. Pravst I. Profeta A. Millard J. (2021). Changes in food consumption during the COVID-19 pandemic: analysis of consumer survey data from the first lockdown period in Denmark, Germany, and Slovenia. Front. Nutr. 8:635859. doi: 10.3389/fnut.2021.635859, PMID: 33763443 PMC7982667

[ref26] Kim H. John D. R. (2008). Consumer response to brand extensions: construal level as a moderator of the importance of perceived fit. J. Consum. Psychol. 18, 116–126. doi: 10.1016/j.jcps.2008.01.006

[ref27] Kim B. K. Zauberman G. Bettman J. R. (2012). Space, time, and intertemporal preferences. J. Consum. Res. 39, 867–880. doi: 10.1086/666464

[ref28] Lei P. Zhang H. Zheng W. Zhang L. (2024). Does sadness bring myopia: an intertemporal choice experiment with college students. Front. Psychol. 15:1235951. doi: 10.3389/fpsyg.2024.1345951, PMID: 38737957 PMC11085738

[ref29] Lerner J. S. Keltner D. (2001). Fear, anger, and risk. J. Pers. Soc. Psychol. 81, 146–159. doi: 10.1037/0022-3514.81.1.146, PMID: 11474720

[ref9001] Leung C. Ho M. K. Bharwani A. A. Cogo-Moreira H. Wang Y. Chun Chow M. S.. (2022). Mental disorders following COVID-19 and other epidemics: a systematic review and meta-analysis. Transl. Psychiatry 12:205. doi: 10.1038/s41398-022-01946-6 35581186 PMC9110635

[ref30] Li X. Wang K. L. Jiang Q. Q. (2024). How to make recommendations on mobile social e-commerce more effective: the role of social features and temporal cues. Inf. Manag. 61:104002. doi: 10.1016/j.im.2024.104002

[ref31] Li H. B. Zheng S. Q. Liu F. Liu W. Zhao R. S. (2021). Fighting against COVID-19: innovative strategies for clinical pharmacists. Res. Soc. Adm. Pharm. 17, 1813–1818. doi: 10.1016/j.sapharm.2020.04.003, PMID: 32278766 PMC7194937

[ref32] Liberman N. Trope Y. (1998). The role of feasibility and desirability considerations in near and distant future decisions: a test of temporal construal theory. J. Pers. Soc. Psychol. 75, 5–18. doi: 10.1037/0022-3514.75.1.5

[ref9002] Martina R. Bernhard S. Eva L. Frey D. (2015). How far does it feel? Construal level and decisions under risk. J. Appl. Res. Mem. Cogn. 4, 256–264. doi: 10.1016/j.jarmac.2014.09.005

[ref33] Motta M. Benegal S. (2023). How pandemic-related changes in global attitudes toward the scientific community shape "post-pandemic" environmental opinion. Public Underst. Sci. 32, 907–925. doi: 10.1177/09636625231167735, PMID: 37204071 PMC10200811

[ref34] Mullainathan A. Shafir E. Zhao J. (2013). Response to comment on "poverty impedes cognitive function". Science 342:1169. doi: 10.1126/science.1246799 24311666

[ref35] National Bureau of Statistics in China (NBSC). (2021). China statistical yearbook (2021). Available online at: https://www.stats.gov.cn/sj/ndsj/2021/indexch.htm

[ref36] Ochi S. Leppold C. Kato S. (2020). Impacts of the 2011 Fukushima nuclear disaster on healthcare facilities: a systematic literature review. Int. J. Disaster Risk Reduct. 42:101350. doi: 10.1016/j.ijdrr.2019.101350

[ref37] Reczek R. W. Trudel R. White K. (2018). Focusing on the forest or the trees: how abstract versus concrete construal level predicts responses to eco-friendly products. J. Environ. Psychol. 57, 87–98. doi: 10.1016/j.jenvp.2018.06.003

[ref38] Sabbaghtorkan M. Batta R. He Q. (2020). Prepositioning of assets and supplies in disaster operations management: review and research gap identification. Eur. J. Oper. Res. 284, 1–19. doi: 10.1016/j.ejor.2019.06.029, PMID: 40478348

[ref39] Schnall A. H. Wolkin A. Bayleyegn T. M. (2018). “Chapter 7: applications: community assessment for public health emergency response” in Disaster epidemiology. ed. Horney J. A. (Atlanta: Academic Press), 75–84.

[ref40] Schwartz A. Eyal T. Tamir M. (2018). Emotions and the big picture: the effects of construal level on emotional preferences. J. Exp. Soc. Psychol. 78, 55–65. doi: 10.1016/j.jesp.2018.05.005

[ref41] Shahbazi M. Bunker D. (2024). Social media trust: fighting misinformation in the time of crisis. Int. J. Inf. Manag. 77:102780. doi: 10.1016/j.ijinfomgt.2024.102780

[ref42] Shan S. Zhao F. Wei Y. Liu M. (2019). Disaster management 2.0: a real-time disaster damage assessment model based on mobile social media data—a case study of Weibo (Chinese twitter). Saf. Sci. 115, 393–413. doi: 10.1016/j.ssci.2019.02.029

[ref43] Siddiqui R. A. Monga A. Buechel E. C. (2018). When intertemporal rewards are hedonic, larger units of wait time boost patience. J. Consum. Psychol. 28, 612–628. doi: 10.1002/jcpy.1019

[ref44] Steiger B. K. Kegel L. C. Spirig E. Jokeit H. (2019). Dynamics and diversity of heart rate responses to a disaster motion picture. Int. J. Psychophysiol. 143, 64–79. doi: 10.1016/j.ijpsycho.2019.06.015, PMID: 31254545

[ref45] Talwar M. Talwar S. Kaur P. Tripathy N. Dhir A. (2021). Has financial attitude impacted the trading activity of retail investors during the COVID-19 pandemic? J. Retail. Consum. Serv. 58:102341. doi: 10.1016/j.jretconser.2020.102341

[ref46] Tisdell C. A. (2020). Economic, social and political issues raised by the COVID-19 pandemic. Econ. Anal. 68:17. doi: 10.1016/j.eap.2020.08.002, PMID: 32843816 PMC7440080

[ref47] Trope Y. Liberman N. (2010). Construal level theory of psychological distance. Psychol. Rev. 117, 440–463. doi: 10.1037/a0018963, PMID: 20438233 PMC3152826

[ref48] Wang Y. C. Guo R. (2024). Tourism e-commerce marketing following live-streaming: consumer behavior and verification psychology. Tour. Rev. 80, 914–927. doi: 10.1108/TR-10-2023-0738

[ref49] Xu L. Yang Z. H. Chen J. H. Zou Z. Y. (2024a). Spatial-temporal heterogeneity of global ports resilience under pandemic: a case study of COVID-19. Marit. Policy Manag. 51, 1655–1688. doi: 10.1080/03088839.2023.2224811

[ref50] Xu S. F. Zhao X. Y. Chen J. (2024b). A temporal approach to online discussion during disasters: applying SIR infectious disease model to predict topic growth and examining effects of temporal distance. Public Relat. Rev. 50:102430. doi: 10.1016/j.pubrev.2024.102430

[ref51] Yuan K. Zheng Y. B. Wang Y. J. Sun Y. K. Gong Y. M. Huang Y. T.. (2022). A systematic review and meta-analysis on prevalence of and risk factors associated with depression, anxiety and insomnia in infectious diseases, including COVID-19: a call to action. Mol. Psychiatry 7, 3214–3222. doi: 10.1038/s41380-022-01638-z PMC9168354 35668158

[ref52] Zaman K. Shah I. A. Ahmad M. (2012). Understanding the relationship between economic growth, employment, income inequality and poverty in Pakistan. J. Soc. Econ. Dev. 1, 1–23.

[ref53] Zhang C. Fan C. Yao W. Hu X. Mostafavi A. (2019). Social media for intelligent public information and warning in disasters: an interdisciplinary review. Int. J. Inf. Manag. 49, 190–207. doi: 10.1016/j.ijinfomgt.2019.04.004

[ref54] Zhang J. Wang T. (2023). Urban resilience under the COVID-19 pandemic: a quantitative assessment framework based on system dynamics. Cities 136:104265. doi: 10.1016/j.cities.2023.104265, PMID: 36883169 PMC9970928

[ref55] Zhang J. Xie C. Liu Y. Q. Li W. T. Huang Q. (2024). Identification of tourists' dynamic risk decision-making: a crisis communication lifecycle perspective. Curr. Issues Tour., 1–18. doi: 10.1080/13683500.2024.2439386

[ref56] Zhao Y. X. Cheng S. X. Yu X. Y. Xu H. L. (2020). Chinese public's attention to the COVID-19 epidemic on social media: observational descriptive study. J. Med. Internet Res. 22:e18825. doi: 10.2196/18825, PMID: 32314976 PMC7199804

