# Supplementary material for: Does declining income caused by the COVID-19 pandemic affect Chinese individuals’ future risky decision-making and intertemporal choices? A construal level perspective
Source: Front Psychol. 2025 Jun 20;16:1584337. doi: 10.3389/fpsyg.2025.1584337 (PMC12231509; doi:10.3389/fpsyg.2025.1584337)
Supplement: Supplementary file 1 [file Supplementary_file_1.docx]

**Appendix 1**

Abbreviations of main variables.

| **IDSC** | **Income decline shock construal Level** | **ST** | **Social Trust** |
| --- | --- | --- | --- |
| HIDSC | High income decline shock construal Level | McT | Medical Trust |
| LIDSC | Low income decline shock construal Level | MeT | Media Trust |
|  |  | CT | Community Trust |
| **AP** | **Anxiety perception about supply shortages** | **HSCB** | **Household saving and consumption behavior** |
| LA | Living supply shortages anxiety perception | LRB | Low risk saving behavior |
| PA | Prevention and control supply shortages anxiety perception | ACB | Advanced consumption behavior |
