# Supplementary material for: Does declining income caused by the COVID-19 pandemic affect Chinese individuals’ future risky decision-making and intertemporal choices? A construal level perspective
Source: Front Psychol. 2025 Jun 20;16:1584337. doi: 10.3389/fpsyg.2025.1584337 (PMC12231509; doi:10.3389/fpsyg.2025.1584337)
Supplement: Supplementary file 2 [file Supplementary_file_2.docx]

**Appendix 2**

Bootstrap test for indirect effect of condition model.

| TPB | Cluster | ST | Mediator：LA | | | | Mediator：PA | | | |
| --- | --- | --- | --- | --- | --- | --- | --- | --- | --- | --- |
|  |  |  | Effect | BootSE | BootLLCI | BootULCI | Effect | BootSE | BootLLCI | BootULCI |
| LRB | X1 | 3.356 | 0.017 | 0.029 | -0.041 | 0.074 | 0.038 | 0.024 | -0.005 | 0.089 |
|  |  | 4.094 | 0.045 | 0.021 | 0.003 | 0.087 | 0.051 | 0.019 | 0.014 | 0.090 |
|  |  | 4.832 | 0.079 | 0.035 | 0.013 | 0.149 | 0.064 | 0.032 | 0.003 | 0.131 |
|  | X2 | 3.356 | 0.024 | 0.021 | -0.015 | 0.067 | 0.033 | 0.017 | 0.001 | 0.070 |
|  |  | 4.094 | 0.059 | 0.019 | 0.023 | 0.097 | 0.064 | 0.018 | 0.030 | 0.099 |
|  |  | 4.832 | 0.102 | 0.035 | 0.034 | 0.172 | 0.103 | 0.035 | 0.036 | 0.172 |
| ACB | X1 | 3.356 | 0.012 | 0.021 | -0.029 | 0.055 | 0.039 | 0.023 | -0.005 | 0.088 |
|  |  | 4.094 | 0.043 | 0.020 | 0.002 | 0.082 | 0.060 | 0.022 | 0.018 | 0.103 |
|  |  | 4.832 | 0.090 | 0.040 | 0.014 | 0.172 | 0.084 | 0.043 | 0.005 | 0.172 |
|  | X2 | 3.356 | 0.018 | 0.016 | -0.011 | 0.052 | 0.033 | 0.017 | 0.002 | 0.069 |
|  |  | 4.094 | 0.057 | 0.019 | 0.021 | 0.095 | 0.075 | 0.020 | 0.036 | 0.115 |
|  |  | 4.832 | 0.115 | 0.041 | 0.036 | 0.198 | 0.134 | 0.045 | 0.046 | 0.221 |
