# Supplementary material for: Does declining income caused by the COVID-19 pandemic affect Chinese individuals’ future risky decision-making and intertemporal choices? A construal level perspective
Source: Front Psychol. 2025 Jun 20;16:1584337. doi: 10.3389/fpsyg.2025.1584337 (PMC12231509; doi:10.3389/fpsyg.2025.1584337)
Supplement: Supplementary file 3 [file Supplementary_file_3.docx]

**Appendix 3**

Bootstrap test for direct effect of Condition model.

|  |  |  | Mediator：LA | | | | Mediator：PA | | | |
| --- | --- | --- | --- | --- | --- | --- | --- | --- | --- | --- |
| TPB | Cluster | ST | Effect | BootSE | BootLLCI | BootULCI | Effect | BootSE | BootLLCI | BootULCI |
| LRB | X1 | 3.3559 | 0.2456 | 0.0815 | 0.0857 | 0.4055 | 0.2275 | 0.0832 | 0.0643 | 0.3906 |
|  |  | 4.0938 | 0.2539 | 0.0539 | 0.1481 | 0.3596 | 0.251 | 0.0548 | 0.1436 | 0.3585 |
|  |  | 4.8316 | 0.2621 | 0.0697 | 0.1254 | 0.3989 | 0.2746 | 0.0707 | 0.1358 | 0.4134 |
|  | X2 | 3.3559 | 0.1484 | 0.0664 | 0.0181 | 0.2787 | 0.139 | 0.0675 | 0.0066 | 0.2713 |
|  |  | 4.0938 | 0.1861 | 0.0503 | 0.0874 | 0.2849 | 0.1814 | 0.0512 | 0.081 | 0.2819 |
|  |  | 4.8316 | 0.2238 | 0.0764 | 0.074 | 0.3737 | 0.2239 | 0.0776 | 0.0716 | 0.3762 |
| ACB | X1 | 3.3559 | 0.1509 | 0.0796 | -0.0053 | 0.3071 | 0.1304 | 0.079 | -0.0246 | 0.2853 |
|  |  | 4.0938 | 0.2139 | 0.0526 | 0.1106 | 0.3171 | 0.2051 | 0.052 | 0.1031 | 0.3071 |
|  |  | 4.8316 | 0.2768 | 0.0681 | 0.1432 | 0.4104 | 0.2798 | 0.0672 | 0.148 | 0.4116 |
|  | X2 | 3.3559 | 0.0147 | 0.0649 | -0.1125 | 0.1419 | 0.0003 | 0.0641 | -0.1254 | 0.1259 |
|  |  | 4.0938 | 0.0931 | 0.0492 | -0.0033 | 0.1896 | 0.0759 | 0.0486 | -0.0195 | 0.1713 |
|  |  | 4.8316 | 0.1716 | 0.0746 | 0.0252 | 0.3179 | 0.1515 | 0.0737 | 0.0069 | 0.2961 |
